# The Anatomical Nature of Dental Paresthesia: A Quick Review

**DOI:** 10.2174/1874210601812010155

**Published:** 2018-02-22

**Authors:** Maha Ahmad

**Affiliations:** Department of Biomedical and Diagnostic Sciences, School of Dentistry, University of Detroit Mercy, Detroit, MI 48208, USA

**Keywords:** Dental paresthesia, Inferior alveolar injections, Anatomy, Pterygomandibular fossa, Anesthetic agents, Dysesthesia

## Abstract

Dental paresthesia is loss of sensation caused by maxillary or mandibular anesthetic administration before dental treatment. This review examines inferior alveolar block paresthesia symptoms, side effect and complications. Understanding the anatomy of the pterygomandibular fossa will help in understanding the nature and causes of the dental paresthesia. In this review, we review the anatomy of the region surrounding inferior alveolar injections, anesthetic agents and also will look also into the histology and injury process of the inferior alveolar nerve.

## 1 INTRODUCTION

A considerable amount of literature examined the complications of inferior alveolar blocks following a dental procedure [1-4]. Allodynia, prolonged anesthesia, paresthesia and dysesthesia are some of these post-treatment complications [5]. There are many speculations and theories examining the possible causes of these complications including paresthesia of lower mandible and/or lower lips [6], which may result from inferior alveolar nerve or lingual nerve injuries following anesthetic injections, anesthetic toxicity [6, 7] caused by multiple injections and/or high concentration of anesthetic agent [2]. There are also other factors that should be taken into consideration including needle gauge, patient and dentist age [8] as well as anatomical variation within the pterygomandibular fossa.

## 2 INFERIOR ALVEOLAR NERVE PARESTHESIA

Paresthesia is defined as altered sensation exhibited as numbness, burning or tingling of patient skin [5]. The etiology of inferior alveolar nerve paresthesia is somewhat unknown, yet may occur following various dental procedures ranging from simple anesthetic injections [6, 9], surgical [10], orthodontic procedures [11]. Third molar extractions [12] and oral pathologies [13] can also cause inferior alveolar paresthesia as well. Endodontic treatment has been associated with paresthesia [14], Froes **et al*.* [15], reported paresthesia as a result of endodontic sealer extrusion that leaked into the mandibular canal.

Inferior alveolar or lingual nerve paresthesia [6, 16] is a complication of inferior alveolar nerve blocks [7]. In some cases, paresthesia can be interpreted as injury to the inferior alveolar or lingual nerve bundle. The majority of cases involving lingual nerve neuropathies (89%) were more frequent following mandibular nerve blocks [6].

There is a wide variety of anesthetic agents used in dental procedures. Articaine is the anesthetic of choice used in many dental practices [9]. Its use is thought to be optimal; as it is proven to be efficient and it is easily diffusible through bone and tissue. The formula most commonly used in US and Canada is Articaine hydrochloride 4% with epinephrine 1:100,000 [17, 18]. Certain anesthetic formulations such as Articaine 4% and Prilcaine 3-4% have been suggested to have a neurotoxic effect causing sensory loss [18] simply because of the higher concentration of the anesthetic [8]. Articaine-related paresthesia ranged as high as 71% [18] and as low as 33% in other studies [19]. This wide range of difference in Articaine-related paresthesia may depend on the method, search queries and the database used in the study [8]. Articaine paresthesia was significantly higher than marketshare paresthesia in majority of studies [8]. However, cell culture experiments and animal studies did not find a higher toxicity of Articaine compared to other anesthetics.

Gaffen and Haas (2009) [17] reported that Articaine had the lion’s share of paresthesia cases (59%) compared to other classes of anesthetic used between 1999 and 2008. This data was supported by a previous study by Haas and Lennon (1995) [18] that indicated that Articaine was used in the majority of non-surgical paresthesia cases in Ontario between 1973 and 1993. Prilocaine came in the second place following Articaine. Tongue paresthesia, then lip paresthesia was the most frequent side effects, with combined tongue and lip paresthesia in a few cases. Taste loss, speech impairment and drooling are symptoms that may accompany oral paresthesia. It is argued that anesthesia could have a neurotoxic effect to the inferior alveolar nerve fibers especially if the needle delivering anesthesia penetrates the neuronal sheath. However, almost half of the cases of inferior alveolar nerve damage exhibit no signs of nerve trauma caused by needle injection [1].

There are numerous studies that present an array of clinical cases of prolonged lingual and mandibular anesthesia [3]. Loss of sensation may resolve in a matter of days, weeks or few months [7, 9]. In a few rare cases there was an unresolved loss of sensation that lasted for more than six months [6]. Brann **et al** (1999) [20] found that patients who underwent general anesthesia before third molar extraction had a five times higher incidence of lingual or inferior alveolar nerve damage.

Third molar extractions are very common procedures in most dental practices. Many complications might result from third molar extractions including bleeding, pain, swelling, dry socket, trismus and paresthesia [12, 21]. The chief postoperative complaint of was numbness [22]. The proximity of the third molar roots to the inferior alveolar nerve might play a role in developing inferior alveolar or lingual nerve damage postoperatively [21]. Many radiological studies focus on the location of the inferior alveolar nerve and its relation with the third molar, subsequently finding that there has been narrowing of the mandibular canal in that region. Thickness of the alveolar bone surrounding the mandibular canal is a crucial in preventing dental paresthesia to avoid perforation into the inferior alveolar nerve especially when performing implant placement [23] or treating periodontal pocket accompanied with alveolar bone loss [24]. However, there are new promising treatments to restore bone loss of the alveolar bone using biodegradable chitosan derivatives [24, 25].

It was reported in 31 out of 134 cases of paresthesia, patients had felt an “electric shock” sensation at the time of anesthetic administration [18] which might be an indication of nerve injury by trauma at the site of injection. Seddon (1943) [26] has attempted to classify nerve injuries based on the severity of the injury. Neurapraxia, the mildest form of nerve injury [5], may lead to mild paresthesia that resolves within a few hours or days. Axonotmesis, the second type of nerve injury which is more severe, is exhibited as severe paresthesia that may take several months to resolve. At this level of nerve injury the nerve bundle is generally intact but there is a small degree of nerve degeneration. Neurotmesis is most severe type of nerve injury where the nerve is completely disrupted. A complete loss of sensory innervation is observed in this case. Sunderland (1978) [27] used a similar classification of neuronal injury as Seddon. Sunderland’s nerve injury scale ranges from grade I to V escalating in terms of severity.

It has been suggested that dental injection complications might be due to neurotoxicity and concentration of anesthetic agent used [8] or simply caused mechanical injury to the nerve by barbed needle [4]. In general local anesthetics including Articaine are considered safe if the dental injection is performed properly. The procedure difficulty, needle gauge and age of the dentist [8] are all factors that should be taken into consideration to avoid inferior alveolar nerve damage.

To understand the nature of dental paresthesia and their relationship with the site of inferior alveolar or mandibular nerve blocks, dentists need to appreciate the anatomy of pterygomandibular fossa which will be described in this next section.

## 3 ANATOMY OF PTERYGOMANDIBULAR FOSSA

Understanding the anatomy of pterygomandibular fossa is crucial for dentists since it is the target space for local anesthetic administration prior dental treatment. Key structures that are present in pterygomandibular fossa include lingual nerve, inferior alveolar nerve and nerve to the mylohyoid. The sphenomandibular ligament and the interpterygoid fascia are also integral structures that define this area [28, 29]. The borders of pterygomandibular fossa are bounded posteriorly by parotid glandular tissue and anteriorly by pterygomandibular raphe made by the union of buccinator and superior constrictor muscles. The lateral border is defined by the mandibular ramus and the medial border is formed by medial and lateral pterygoid muscles. The inferior alveolar nerve is one of the most important branches of the mandibular branch of the trigeminal nerve. Before its entry to the mandibular foramen near the lingula of the mandible; it gives off their well-known branch the mylohyoid nerve supplying both the mylohyoid and anterior belly of digastric muscles. Toward the end of the mandibular foramen; the inferior alveolar nerve continues as the mental nerve emerging from the mental foramen and the incisive nerve continuing in course anteriorly. Both of these nerve branches are sensory, the mental nerve supplies the skin of chin and oral mucosa while the incisive nerve is responsible for providing sensory innervation for premolar, canines and incisors. The inferior alveolar nerve is also accompanied with inferior alveolar artery and vein. Inferior alveolar artery is a branch of the mandibular artery, although it has been reported that it can branch off the external carotid artery [30]. Another study of 56 hemisected cadavers [29] head analyzed crucial structures in pterygomandibular fossa indicating an average of two inferior alveolar veins per specimen. In addition to that the inferior alveolar nerve was found to be anterior to inferior alveolar vasculature in most specimens.

There is a considerable amount of literature regarding anatomical variations in pterygomandibular fossa especially the inferior alveolar nerve [31]. Variant of the inferior alveolar nerve has been a classified into extra-osseous and intra-osseous multiple branches of the nerve. The presence of extra-ossesous branches has been concurrent with the presence of multiple accessory foramina in the mandible.

Lingual nerve injury has been reported in some cases of inferior alveolar nerve blocks [6, 16]. In study by Morris **et al**., 2010 [16] simulated inferior alveolar injections were performed in cadavers head to estimate probability of lingual nerve trauma associated with inferior alveolar nerve blocks. The location of the lingual nerve was highly variable; although about 96% of the injections were made lateral to the lingual nerve. A small fraction 4.5% of these simulated injections has actually penetrated the lingual nerve.

The location of lingual nerve in cadavers’ study done by Kiesselbach and Chamberlain, 1984 [32] was found to be in direct contact with lingual crest in majority of cadavers (62%) and superior to the lingual crest in only 17.6% of cadavers dissected in the study.

## 4 HISTOLOGICAL FEATURES OF INJURED NERVE

Inferior alveolar nerve has a structure of any typical peripheral nerve with three layers of epineurium, perineurium and endoneurium [5] that surrounds peripheral nerve bundles. The outermost and thickest layer is the epineurium and the innermost slender layer immediately rapping itself around the nerve is the endoneurium. The endoneurium is the most intimate layer surrounding individual neuron following the myelin sheath. It has been argued that there is different number of fascicles surrounding inferior alveolar nerve thus causing some patient to be predisposed to paresthesia after a dental procedure. The number of fascicles surrounding individual nerves might be an anatomical variation that has to be examined closely. Pogrel *et al* 2003 [1] have found an average of 20 fascicles surrounding the lingual nerve at lingula the number of fascicles around the lingual number ranged between 7 and 39, while the numbers of fascicles around the inferior alveolar nerve at lingula varied between 3 and 14 with an average of 7.2 fascicles.

Different imaging techniques have been used to determine the level of neuronal injury [33]. Panoramic radiography, computed tomography, magnetic source imaging and ultrasonography are all different types of imaging that were examined in recent study to evaluate post injury status of the inferior alveolar nerve. Magnetic Resonance Neurography (MRN) has the ability to distinguish injured nerve fiber from intact nerves. Injury to nerve during the anesthesia could cause hemorrhage within nerve fascicles that would cause pressure on the nerve and eventually would interfere with the nerve function. The nerve diameter would increase as result of nerve injury [33].

## CONCLUSION

The etiology of paresthesia following dental procedure is still mysterious. Anatomical variations concerning the location of the lingual and inferior alveolar nerve, and variant branches of the nerve or the presence of multiple mandibular canals might be a possible cause for dental paresthesia. The histology of the lingual and inferior alveolar nerve and the number of fascicles surrounding these nerves are also very important factors in understanding the reasons behind paresthesia affecting these nerves.
